# Promoter Hypermethylation Mediated Downregulation of FBP1 in Human Hepatocellular Carcinoma and Colon Cancer

**DOI:** 10.1371/journal.pone.0025564

**Published:** 2011-10-19

**Authors:** Mingquan Chen, Jianbin Zhang, Ning Li, Zhiping Qian, Mengqi Zhu, Qian Li, Jianming Zheng, Xinyu Wang, Guangfeng Shi

**Affiliations:** 1 Department of Infectious Diseases, Huashan Hospital, Fudan University, Shanghai, China; 2 School of Medicine, Institute of Immunology, Zhejiang University, Hangzhou, China; The University of Hong Kong, China

## Abstract

FBP1, fructose-1,6-bisphosphatase-1, a gluconeogenesis regulatory enzyme, catalyzes the hydrolysis of fructose 1,6-bisphosphate to fructose 6-phosphate and inorganic phosphate. The mechanism that it functions to antagonize glycolysis and was epigenetically inactivated through NF-kappaB pathway in gastric cancer has been reported. However, its role in the liver carcinogenesis still remains unknown. Here, we investigated the expression and DNA methylation of FBP1 in primary HCC and colon tumor. FBP1 was lowly expressed in 80% (8/10) human hepatocellular carcinoma, 66.7% (6/9) liver cancer cell lines and 100% (6/6) colon cancer cell lines, but was higher in paired adjacent non-tumor tissues and immortalized normal cell lines, which was well correlated with its promoter methylation status. Methylation was further detected in primary HCCs, gastric and colon tumor tissues, but none or occasionally in paired adjacent non-tumor tissues. Detailed methylation analysis of 29 CpG sites at a 327-bp promoter region by bisulfite genomic sequencing confirmed its methylation. FBP1 silencing could be reversed by chemical demethylation treatment with 5-aza-2′-deoxycytidine (Aza), indicating direct epigenetic silencing. Restoring FBP1 expression in low expressed cells significantly inhibited cell growth and colony formation ability through the induction of G2-M phase cell cycle arrest. Moreover, the observed effects coincided with an increase in reactive oxygen species (ROS) generation. In summary, epigenetic inactivation of FBP1 is also common in human liver and colon cancer. FBP1 appears to be a functional tumor suppressor involved in the liver and colon carcinogenesis.

## Introduction

Hepatocellular carcinoma (HCC) remains the third leading cause of cancer death worldwide and the second most common malignancy in China. The molecular pathogenesis of HCC remains largely unknown. It has been hypothesized that development and progression of HCC are the consequence of cumulative genetic and epigenetic events. CpG hypermethylation acts as an alternative mechanism to gene inactivation, and it is now recognized as an important mechanism during tumor initiation and progression, including liver cancer [1], [2]. It is important and intriguing to identify new genes silenced by promoter hypermethylation in liver cancer because aberrant tumor suppressor gene (TSG) hypermethylation is both a mechanism and a biomarker for tumorigenesis [3].

FBP1, fructose-1,6-bisphosphatase 1, a gluconeogenesis regulatory enzyme, catalyzes the hydrolysis of fructose 1,6-bisphosphate to fructose 6-phosphate and inorganic phosphate. Fructose-1,6- diphosphatase deficiency is associated with hypoglycemia and metabolic acidosis. It was reported [4] that FBP1 which functions to antagonize glycolysis was downregulated through NF-kappaB pathway in Ras-transformed NIH3T3 cells. NF-kappaB functions downstream of Ras to promote epigenetic downregulation of FBP1. Inhibition of NF-kappaB reversed promoter methylation-mediated FBP1 downregulation. Moreover, promoter methylation of FBP1 was relevant to gastric carcinogenesis, for example, gender, TNM stages and survival rate were significantly associated with FBP1 promoter methylation. Besides, it was studied [5] that FBP1 promoter methylation contributes to the decreased expression of FBPase in breast cancer. When non-malignant and malignant tissue samples from the same patient were compared a correlation between an increase of FBPase promoter methylation and a decrease of FBPase mRNA levels was observed. However, no findings are shown for FBP1 epigenetic inactivation in the liver carcinogenesis.

Reactive oxygen species (ROS), chemically-reactive molecules containing oxygen, including oxygen ions and peroxides, are the key mediators of cellular oxidative stress and redox dysregulation involved in cancer initiation and progression. It is now widely accepted that constitutively elevated levels of cellular oxidative stress and dependence on mitogenic and anti-apoptotic ROS-signaling in cancer cells are involved in the carcinogenesis. Paradoxically, apart from being involved in proliferative, anti-apoptotic, metastatic, and angiogenic signaling, ROS may also exert cytotoxic and proapoptotic functions that would limit tumorigenicity and malignant progression [6], [7]. FBP1 is a multifunctional protein that is, in addition to its function in gluconeogenesis, involved in ROS production after treatment with MMS or in chronologically aged cells [8]. In the absence of FBP1 cells survive MMS treatment better and even proliferate after long-term treatment. This could be the result of reduced production of ROS observed in the ΔFBP1 mutant in response to the alkylating agent methylmethane sulfonate (MMS) treatment and aging. Lack of FBP1 improved surviving of aged cells in full medium and resulted in a delayed induction of ROS production. Overexpression of FBP1 reduced the ability to form colonies even of untreated cultures, shortened life span and increased the induction of the RNR2 promoter after MMS treatment.

Here, we demonstrated that FBP1 underwent promoter CpG hypermethylation-associated silencing in human liver and colon cancer. The analysis of liver and colon cancer cell lines and tissues showed that FBP1 hypermethylation was a common event in human liver and colon cancer. We also showed that FBP1 functions as a TSG to suppress cancer cell growth through the induction of G2-M phase cell cycle arrest and an increase in ROS generation levels. Epigenetic changes in cellular redox homeostasis and ROS levels will affect viability through redox modulation of the mitochondrial permeability transition pore opening leading to cytochrome C release, apoptosome assembly and activation of executioner caspases, which is supportive for ROS-directed cancer chemotherapeutics.

## Materials and Methods

### Cancer Cell Lines, Primary Tumor Tissues and RNA/DNA Extraction

Nine human liver cancer cell lines (HepG2, Hep3B, Huh7, HCC-LM3, BEL-7402, SMMC-7721, Sk-Hep1, MHCC-97H and MHCC-97L) were used for HCCs analysis. One immortalized normal human liver cell lines L02 was used as “normal” controls for HCCs analysis. Six human colon cancer cell lines (HT29, SW480, SW620, HCT116, LoVo and RKO) were used for colon cancer analysis and human normal adult colon tissues were used as “normal” controls. All of the cell lines were obtained from American Type Culture Collection (ATCC, Manassas, Va., USA). Unless specifically indicated, cells were cultured in DMEM medium (Invitrogen, Carlsbad, Calif., USA) supplemented with 10% fetal bovine serum at 37°C with 5% CO_2_ and 95% humidity. For pharmacological demethylation, cells were treated with 5 uM 5-aza-2′-deoxycytidine (Aza) (Sigma, St. Louis, Mo., USA) for 3 consecutive days. Culture medium was changed every 24 hours. An equivalent concentration of vehicle (DMSO) was used as the control.

Primary HCCs were obtained from liver cancer patients in Huashan Hospital at the time of surgery; matched non-tumor samples from liver cancer patients were also obtained at least 2 cm distant from the tumor. Non-tumor portions were trimmed off from the frozen tumor blocks and the selected tumor areas had more than 80% tumor cells as confirmed by histology. Human gastric, colon tumor tissues and normal adult colon tissues were provided by Chinese Medicine Hospital of Zhejiang. All patients gave informed consent for obtaining the study specimens.

Total RNA and genomic DNA were extracted using Trizol reagent (Invitrogen) according to the manufacturer's instructions. Total RNA and DNA concentrations were quantified by NanoDrop 1000 (Nanodrop, Wilmington, Del., USA).

### Real-Time RT-PCR

A reverse transcription reaction was performed using 1 ug of total RNA with High Capacity cDNA Reverse Transcription kit (Applied Biosystems, Foster City, Calif., USA). The mRNA expression levels of FBP1 were determined by Real-Time RT-PCR using SYBR Green Master Mix Kit and ABI 7500 Real-Time RT-PCR System (Applied Biosystems, Foster City, Calif., USA). Glyceraldehyde-3- phosphate dehydrogenase (GAPDH) was used as an internal control of RNA integrity. Real-Time RT-PCR was performed in triplicate. Primers used for FBP1 were: FBP1-F 5′-ATCCCCTTGATGGATCTTCC-3′ and FBP1-R 5′-TCCAGCATGAAGCAGTTGAC-3′ (208 bp product).

### Bisulfite Treatment of DNA and Methylation-Specific PCR

Genomic DNA was bisulfite-treated with Zymo DNA Modification Kit (Zymo Research, Orange, Calif., USA) according to the protocol provided. Methylation-specific PCR (MSP) was carried out for 40 cycles with annealing temperature at 60°C. Methylation-specific primers were: FBP1-MF 5′-TGAAGATTTAAGTAGGCGGAGTC-3′ and FBP1-MR 5′-ATAAACACTAACCG CAAATACGAA-3′, and unmethylation-specific primers were: FBP1-UF 5′-GAAGATTTAAGTA GGTGGA GTTGTG-3′ and FBP1-UR 5′-CTAACAAAAAAACTAACAAACCAAC-3′.

### Bisulfite Genomic Sequencing

Bisulfite-treated genomic DNA was amplified using bisulfite genomic sequencing (BGS) primers, FBP1-BF: 5′-TGATTTTGGAGGAAATAGTTATAGTTTTT-3′ and FBP1-BR: 5′-TAATACAACTAAACCAAACCAAAC-3′. PCR products were purified with Illustra GFX™PCR and gel band purification kit (GE Healthcare Life Science, Uppsala, Sweden) and cloned into pCR4-TOPO vector for sequencing (Invitrogen). At least four colonies were randomly chosen for plasmid extraction and sequencing analysis using the ABI PRISM BigDye Terminator Cycle Sequencing Kit in the ABI 3100 sequencer (Applied Biosystems).

### Construction of FBP1 Expressing Vector

The FBP1 expressing vector was constructed by cloning of the full-length FBP1 open reading frame into mammalian expression vector pcDNA3.1. The full-length FBP1 open reading frame was amplified from normal stomach cDNA using high-fidelity PFU DNA polymerase (Invitrogen). The primers used for cloning were FBP1-CF: 5′-CGGAATTCCGATGGCTGACCAGGCGCCCTTCGA-3′ and FBP1-CR: 5′-GCAAGCTTCCTGGGCAGAGTGCTTCTCATACACC-3′ accomp- anied with double enzyme cutting sites ECoRI and Hind III. The sequence and orientation of the insert were confirmed by DNA sequencing.

### Colony Formation Assay

Human liver cancer cells transiently transfected with pcDNA3.1 empty vector or pcDNA-FBP1 expressing vector were used for the monolayer colony formation assay to evaluate cellular growth in vitro. Cells were cultured overnight in a 12-well plate (5×10^5^ cells/well) and transfected with pcDNA3.1 empty vector or pcDNA-FBP1 expressing vector using FuGENE 6 (Roche Applied Science, Mannheim, Germany). Forty-eight hours later, the transfectants were replated in triplicate and cultured for 10∼15 days in complete DMEM medium containing G418 (400 ug/ml). Surviving colonies were stained with Gentian Violet after methanol fixation and visible colonies (≥50 cells) were counted. The experiments were repeated three times.

### Cell Growth Assay

Cells growth were determined by a non-radioactive proliferation assay based on the ability of metabolically active cells to convert 3-(4,5-dimethylthiazol-2-yl)-5(3-carboxymethonyphenol)-2 -(4-sulfophenyl)-2H-tetrazolium (MTS) (Promega, Madison, WI, USA) into formazan. Surviving cells after G418 selection were replated into a 96-well plate with different cell number and cultured in complete DMEM medium containing G418 (400 ug/ml) for another 72 hours. The quantity of formazan was measured as the above. The quantity of formazan was measured at 490 nm absorbance after one hour incubation with CellTiter 96 AQueous One Solution Reagent following instructions provided.

### Cell Cycle Analysis and Determination of ROS Production

Surviving cells after G418 selection were trypsinized, washed in phosphate-buffered saline, and fixed in ice-cold 70% ethanol–phosphate-buffered saline. After washing out ethanol, the fixed cells were treated with 0.01% RNase (10 mg/ml, Sigma, St. Louis, MO, USA) for 10 min at 37°C and then stained with 0.05% propidium iodide for 20 min at 4°C in dark. The cell cycle distribution was determined using a FACScan flow cytometry (Becton Dickinson, Mountain View, CA, USA) and analyzed with Modfit software (Phoenix, San Diego, CA, USA). Cells undergoing apoptosis were detected as sub-G1 population because of loss of fragmented DNA.

ROS levels were measured by using 2′-7′-Dichlorodihydrofluorescein diacetate (DCFH-DA) (Invitrogen) in a flow cytometry assay as described [9].

### Statistic Analysis

The results were expressed as mean ± standard deviation (SD). Student *t* test was used to compare the differences of FBP1 expression on the effect of colony formation and cell proliferation. All statistical calculations were done using SPSS version 11.0 for windows (SPSS, Inc., Chicago, IL). Value of *P*<0.05 was taken as statistical significance.

## Results

### Downregulation of FBP1 in Human Liver and other Digestive Cancers

**Figure 1:**
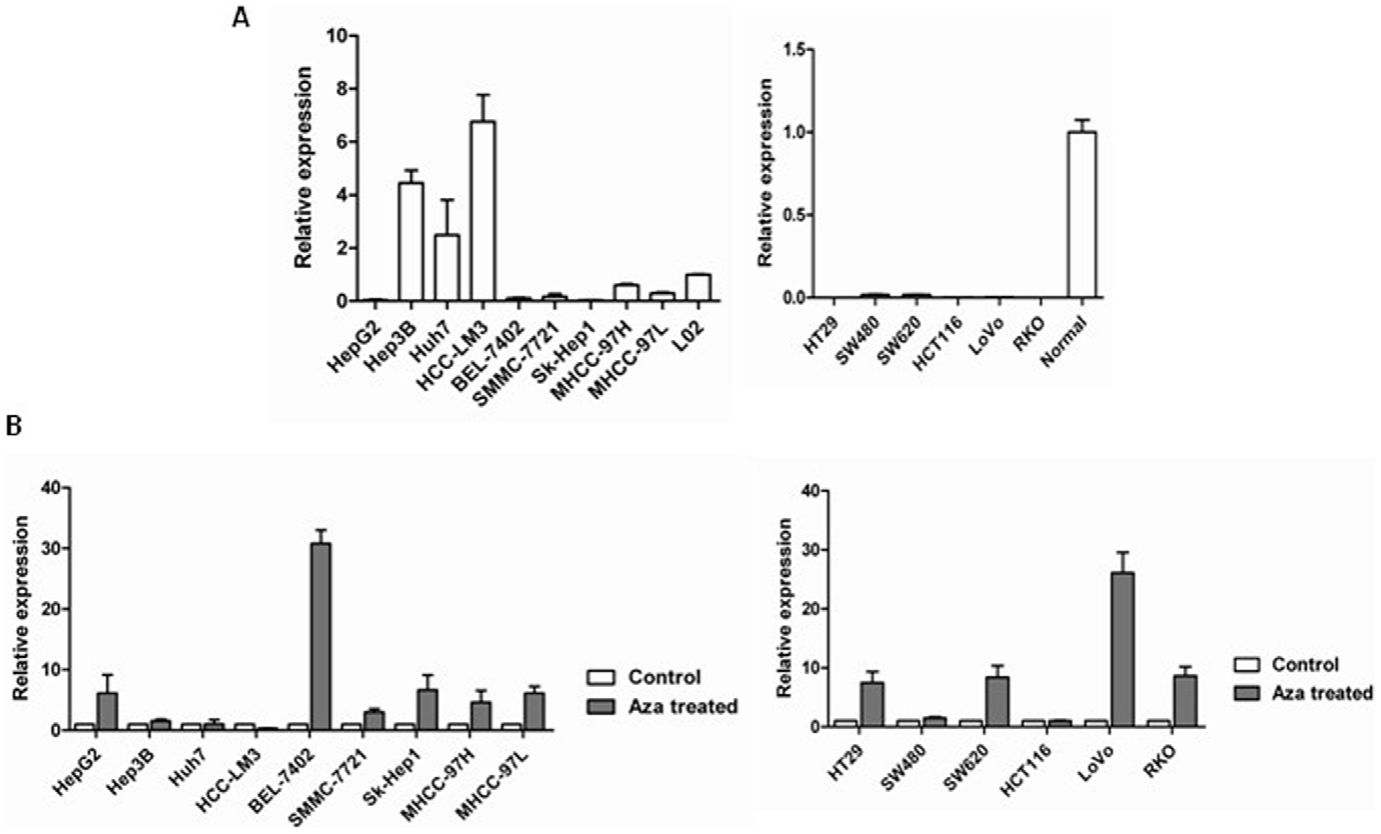
Pharmacological demethylation reversed FBP1 downregulation in human liver and other digestive cancers. (**A**) FBP1 expression in human liver and colon cancer cell lines and normal controls were determined by Real-Time RT-PCR. GAPDH was used to normalize the template amount. (**B**) Relative FBP1 expression before and after Aza treatment were determined by Real-Time RT-PCR. GAPDH was used to normalize the template amount. It was shown as average fold changes ± SD.

The expression of FBP1 was dramatically downregulated in 66.7% (6/9) human liver cancer cell lines including HepG2, BEL-7402, SMMC-7721, Sk-Hep1, MHCC-97H and MHCC-97L (Figure 1A) when compared to human normal liver cells Lo2. Similarly, downregualtion of FBP1 was also found in 100% (6/6) human colon cancer cell lines including HT29, SW480, SW620, HCT116, LoVo and RKO (Figure 1A) when compared to human normal adult colon tissue. In order to know whether downregulation of FBP1 should be attributed to DNA methylation, the expression of FBP1 in human liver and colon cancer cell lines before and after Aza treatment were determined by Real-Time RT-PCR. The expression of FBP1 was significantly upregulated in liver cancer cell lines HepG2, BEL-7402, SMMC-7721, Sk-Hep1, MHCC-97H and MHCC-97L (6/9, 66.7%) after Aza treatment (Figure 1B) and also in colon cancer cell lines HT29, SW620, LoVo and RKO (4/6, 66.7%) (Figure 1B), indicating that FBP1 is likely downregulated through promoter hypermethylation in human liver and colon cancer.

### Methylation of FBP1 Promoter in Human Liver and Colon Cancers

**Figure 2:**
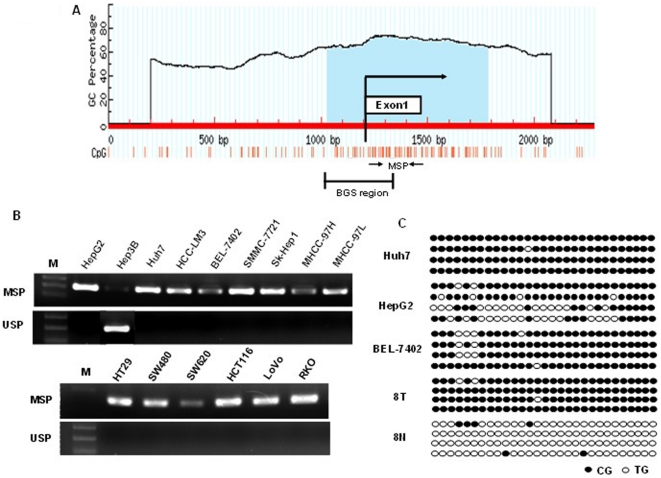
Methylation of FBP1 promoter in human liver and colon cancer. (**A**) Schematic structure of the FBP1 CGI, with the exon 1 and MSP and BGS region indicated. Each short vertical line represents one CpG site. The position of MSP primers were marked as arrows. The methylation status of the FBP1 CGI was analyzed by MSP (**B**) and BGS (**C**). MSP = Methylation-specific PCR; USP = Unmethylation-specific PCR. For BGS in (**C**), each circle indicates one CpG site and circles filled in black represent methylated CpG sites. One row of circles represents a single colony.

Indeed one typical CpG Islands (CGI) were found around FBP1 exon 1 using the following criteria: GC content >55%, Obs CpG/Exp CpG >0.65, and length >500 bp (Figure 2A). The methylation status of this CGI in human liver and colon cancer cells was determined by MSP. As shown in Figure 2B, methylation of FBP1 promoter was readily detected in liver cancer cell lines HepG2, HuH7, HCC-LM3, BEL-7402, SMMC-7721, Sk-Hep1, MHCC-97H and MHCC-97L, and human colon cancer cell lines HT29, SW480, SW620, HCT116, LoVo and RKO. Besides, BGS also revealed that FBP1 promoter was heavily methylated in HepG2, Huh7, BEL-7402 cells and 8T tumor tissue, and no methylation was dedtected in 8N adjacent non-tumor tissue (Figure 2C).

### Downregulation and Promoter Hypermethylation of FBP1 in Human Primary Tumor Tissues

**Figure 3:**
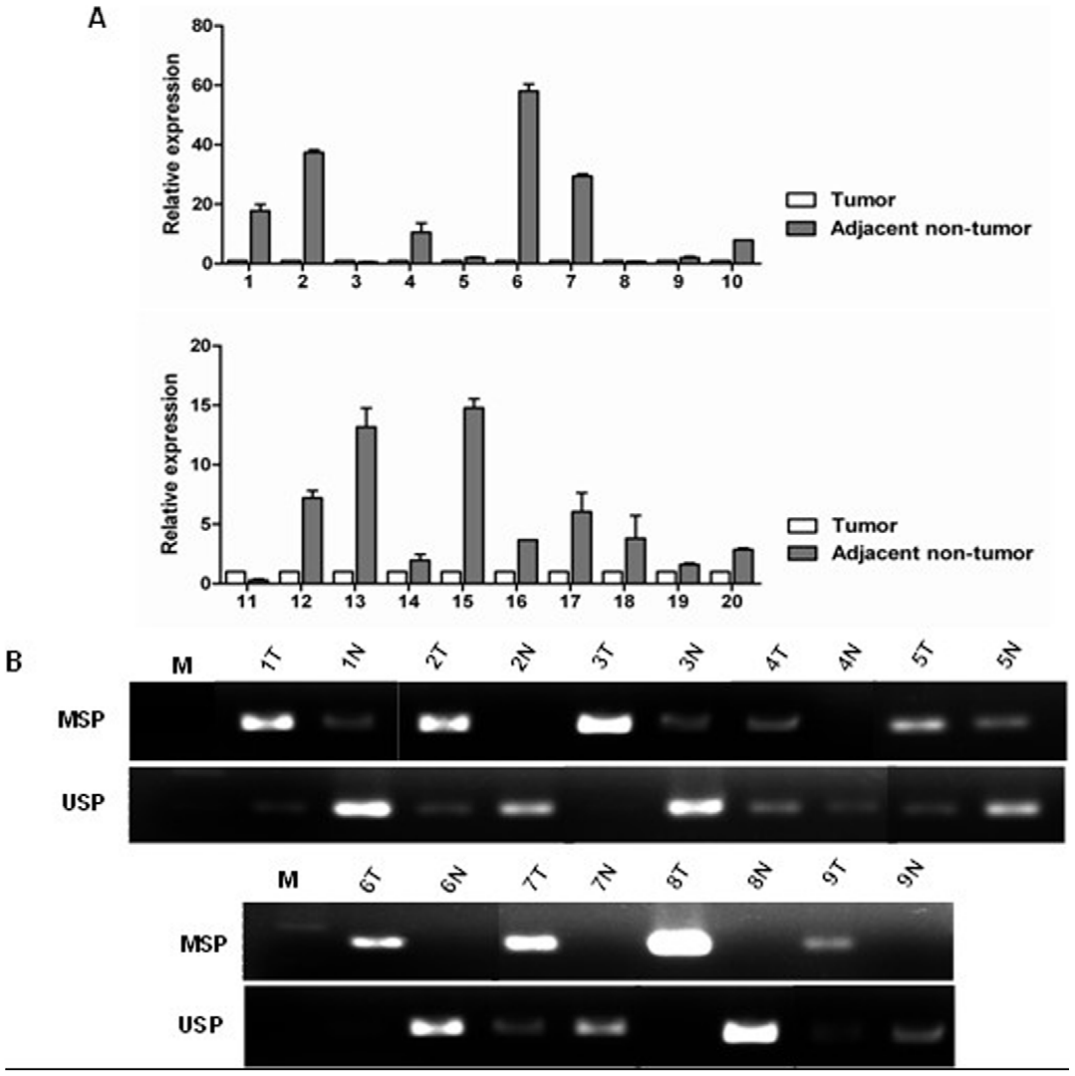
FBP1 gene was downregulated and hypermethylated in primary tumor tissues. (**A**) The expression of FBP1 in liver, gastric and colon tumor tissues and adjacent non-tumor tissues were determined by Real-Time RT-PCR. 1∼10 T/N: liver cancer, 11–15 T/N: gastric cancer, 16∼20 T/N: colon cancer. (**B**) The methylation status of FBP1 promoter in primary liver, gastric and colon tumor tissues and adjacent non-tumor tissues was detected by MSP. Representative results were shown. 1∼3 T/N: liver cancer, 4∼5 T/N: colon cancer, 6∼9 T/N: gastric cancer. “T” indicates tumor tissues and “N” represents adjacent non-tumor tissues.

To further confirm the relevance of FBP1 promoter CGI hypermethylation in mediating its silencing in human liver, gastric and colon cancer, FBP1 expression and promoter methylation in primary hepatocellular carcinoma, gastric and colon tumor tissues and adjacent non-tumor tissues were analyzed by Real-Time RT-PCR and MSP, respectively. FBP1 expression was significantly downregulated in 80% (8/10) human liver tumor tissues, 100% (5/5) in gastric and 80% (4/5) colon tumor tissues (Figure 3A) when compared with adjacent non-tumor tissues. In addition, promoter methylation was frequently detected by using MSP in tumor samples but not non-tumor tissues (Figure 3B), indicating that downregulation of FBP1 was involved in the carcinogenesis of human liver and other digestive cancers.

### FBP1 Overexpression Reduced Cancer Cell Colony Formation Abilities and Inhibited the Growth of Liver and Colon Cancer Cells

**Figure 4:**
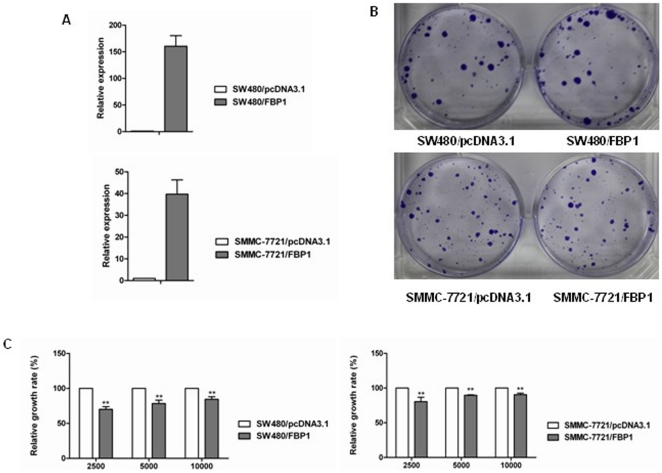
FBP1 overexpression reduced liver cancer cell colony formation abilities. (**A**) FBP1 expression level in the transfected SW480 and SMMC-7721 cells was confirmed by Real-Time RT-PCR. (**B**) The effect of ectopic FBP1 expression on liver cancer cell growth was investigated by the monolayer colony formation assay. The scanned colony formation in six-well plates was shown in the panel.. (**C**) The growth of liver and colon cancer cell lines (SW480 and SMMC-7721) with and without FBP1 expression was determined with MTS cell growth assay. Stable expression of FBP1 suppressed the growth of liver and colon cancer cells. The results were shown as values of mean ± SD. P values were calculated using Student's t-test (* p<0.05).

The effect of exogenous FBP1 expression on the growth of human liver cancer cells was investigated by a monolayer colony formation assay. To further investigate the potential role of FBP1 in tumor suppression, liver cancer cells SMMC-7721 and colon cancer SW480 was transfected with pcDNA-FBP1 expressing vector and the cell colony formation ability was examined under the selection of G418. Compared with control cells transfected with empty vector pcDNA3.1, cancer cells transfected with pcDNA-FBP1 expressing vector showed decreased colony formation ability (Figure 4B). These data suggest that FBP1 may play a role in tumor suppression. Besides, FBP1 stable expression levels in those cells including SW480 and SMMC-7721 was also confirmed by Real-Time RT-PCR as shown in Figure 4A.

When pcDNA-FBP1 was transfected into SW480 and SMMC-7721 cells, the growth inhibitory function of FBP1 in these cells was confirmed by MTS cell growth assay. PcDNA-FBP1 expressing vector was transfected into these cells, the growth speed of human liver and colon cancer cells after FBP1 overexpression was dramatically reduced in MTT cell growth assay (p<0.05; Figure 4C), showing a growth-suppressive effect of FBP1 on cancer cells.

### Ectopic Expression of FBP1 Induced G2-M Phase Arrest

**Figure 5:**
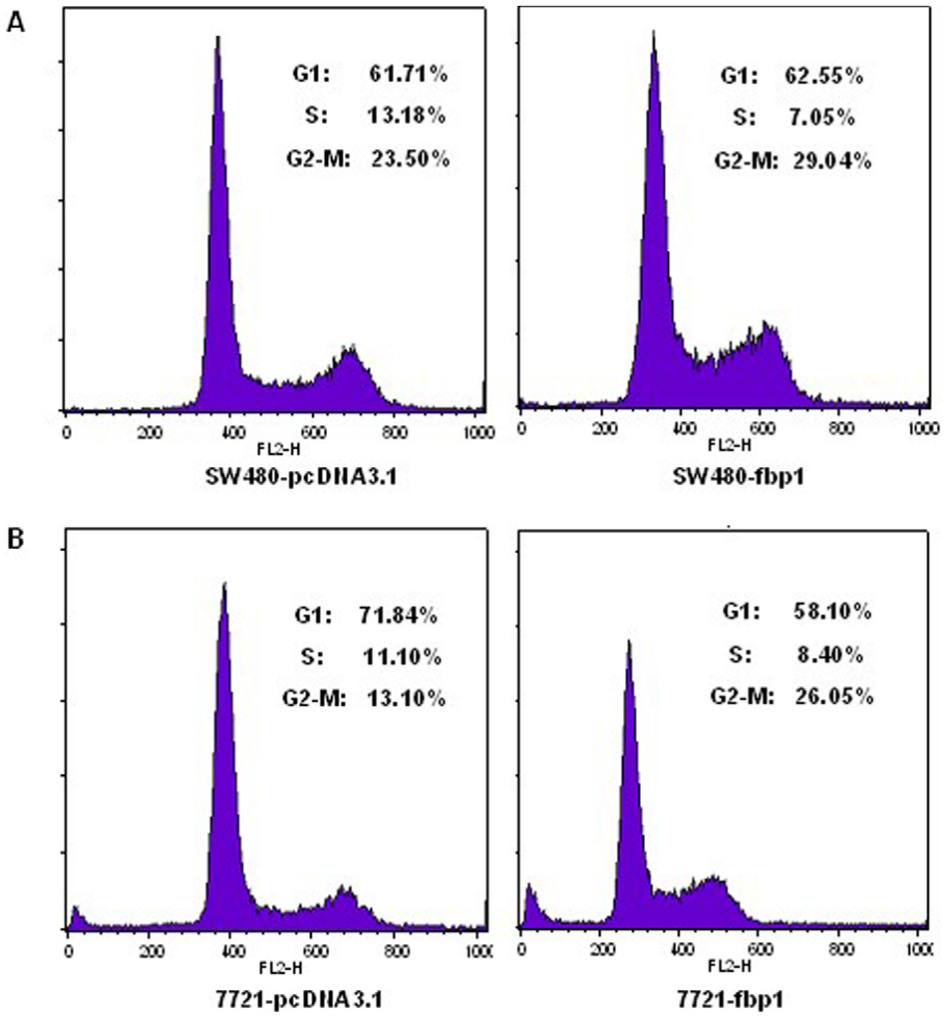
Effect of FBP1 on the cell cycle of liver and colon cancer cell line. The cell cycle distribution of liver and colon cancer cell line (SW480 and SMMC-7721) with and without FBP1 expression was evaluated by flow cytometry analysis. (**A**) and (**B**) Representative fluorescence -activated cell sorting analysis of cancer cells transfected with or without FBP1.

To determine the molecular mechanism by which FBP1 suppressed colony formation and cell proliferation, we investigated the effect of FBP1 on cell cycle distribution. After propidium iodide staining, fluorescence-activated cell sorting analysis of FBP1 overexpressed SW480 and SMMC-7721 cells revealed an increase in the number of G2-M phase cells and a decrease in the number of S phase cells (Figure 5A and 5B).

### FBP1 Overexpression Increased Intracellular ROS Production

**Figure 6:**
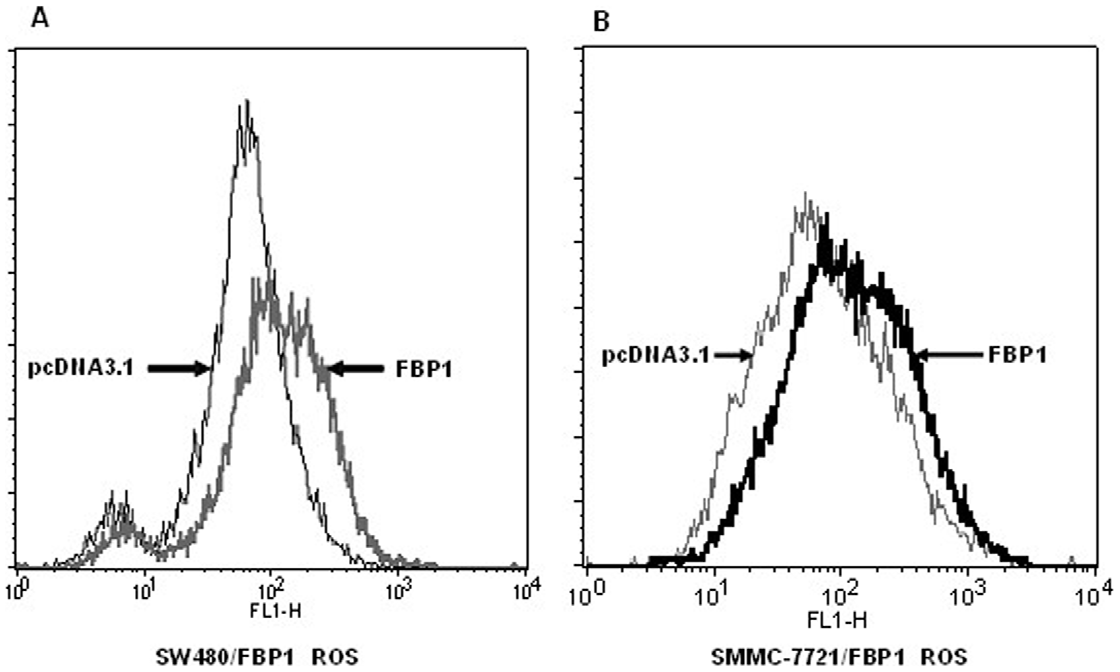
Exogenous FBP1 increased ROS levels in liver and colon cancer cells. The effect of ectopic FBP1 expression on oxidative stress was determined by flow cytometry assay as shown in (**A**) and (**B**). Stable cells were stained with DCFH-DA and then the redox state of cells was measured by flow cytometry.

The ROS levels were measured in cancer cells untransfected and transfected with pcDNA-FBP1 expressing vector. Exogenous FBP1 expression clearly increased intracellular ROS levels in SMMC-7721 and SW480 cells (Figure 6A and 6B). Therefore, FBP1 could increase ROS production which exert cytotoxic and proapoptotic functions and limit tumorigenicity and malignant progression.

## Discussion

More and more novel tumor suppressor genes have been found to be inactivated by promoter hypermethylation. Promoter methylation was thus proposed as an important marker for the identification of novel tumor suppressor genes. In the current study, we identified that FBP1 was frequently down-regulated in 66.7% HCC cell lines and 100% colon cancer cell lines (Figure 1A), and in 80% primary HCCs, 100% gastric tumors and 80% colon tumors (Figure 3A). Hypermethylation was further detected in 66.7% HCC cell lines and 100% colon cancer cell lines (Figure 2B), and also in primary tumor tissues (Figure 3B). In contrast, FBP1 hypermethylation was hardly detected in normal liver cell lines and occasionally in paired adjacent non-tumor tissues, suggesting an important role of FBP1 in the pathogenesis of liver and other digestive cancers. We further demonstrated that treatment with the demethylation reagent Aza upregulated FBP1 expression in lowly expressed cancer cells (Figure 1B) and the methylation status was verified by genomic sequencing, indicating that DNA hypermethylation mediated FBP1 inactivation.

In cancer, the dynamics of genetic and epigenetic gene silencing are very different. Somatic genetic mutation leads to a block in the production of functional protein from the mutant allele. If a selective advantage is conferred to the cell, the cells expand clonally to give rise to a tumor in which all cells lack the capacity to produce protein. In contrast, epigenetically mediated gene silencing occurs gradually. It begins with a subtle decrease in transcription, fostering a decrease in protection of the CpG island from the spread of flanking heterochromatin and methylation into the island. This loss results in gradual increases of individual CpG sites, which vary between copies of the same gene in different cells. For example, higher level of FBP1 was detected in Huh7 and HCC-LM3 cells with high methylation level in the promoter region (Figure 1A and 2B). Although promoter methylation frequently inactivated FBP1 in human liver and colon cancer cell lines, we cannot exclude the involvement of other mechanisms responsible for the FBP1 downregulation, such as defects in histone remodeling. For instance, in Hep3B, Huh7, HCC-LM3, SW480 and HCT116 cells, FBP1 promoter failed to be fully upregulated after Aza treatment (Figure 1B).

In addition, to serve as a new marker to define novel tumor suppressor genes, promoter hypermethylation can also be used as a sensitive marker for cancer diagnosis and prognosis prediction [10], [11]. Unfortunately, for lack of large numbers of tumor tissues, we couldn't test FBP1 methylation in abundant tissues and further analyze its association with clinical characteristics, such as age, gender, tumor grade and survival rate. We had to determine FBP1 expression and methylation status in only 10 pairs of primary HCCs, 5 pairs of gastric tumor tissues and 5 pairs of colon tumor tissues (Figure 3), suggesting that FBP1 functions as a novel tumor suppressor candidate downregulated through promoter hypermethylation in liver and colon cancer. This is also the first report to show that FBP1 is epigenetically silenced in human liver and colon carcinogenesis. Once FBP1 promoter methylation was detected at a high frequency in primary carcinoma tissues but not non-tumor normal gastric tissues, FBP1 promoter methylation may be a potential biomarker for cancer diagnosis.

As is shown in Figure 4, restoration of FBP1 expression markedly suppressed cancer cells growth. However, the underlying molecular mechanism responsible for FBP1 functions as a tumor suppressor remains unknown. It has been verified [4] that FBP1 functions to antagonize glycolysis. As is well known, cancer cells have a higher rate of aerobic glycolysis, but not oxidative phosphorylation. Fructose-1,6-bisphosphate is one of the most important intermediates in glycolysis and its level is mainly controlled by fructose-6-phosphate kinase and fructose-1,6- bisphosphatase. It was found that the production of lactate after FBP1 expression was significantly reduced, demonstrating the suppression of aerobic glycolysis by FBP1. Besides, cell cycle checkpoints are important control mechanisms that ensure the proper execution of cell cycle events. The growth suppression induced by ectopic FBP1 expression seems to be caused by cell cycle arrest since the numbers of cells with cell cycle blockage (G2-M phase arrest) were increased after FBP1 re-expression (Figure 5).

For a long time, ROS were considered oncogenic since it was implicated in cancer progression and metastasis. Persistent oxidative stress has been associated with breast carcinoma and many epithelial cancers such as colon and neck cancers [12]. However, numerous studies have demonstrated the causative involvement of ROS formation in the mediation of cancer cell apoptosis induced by various standard chemotherapeutic agents including paclitaxel, cisplatin, bortezomib and etoposide. Remarkably, it has been demonstrated that cisplatin apoptogenicity depends on formation of ROS and occurs independent of nuclear DNA damage, suggesting that apoptogenic oxidative stress is the crucial mechanism of cisplatin-induced cancer cell death [13]. In addition to causing cell cycle arrest at the S phase, importantly in this study, the growth inhibitory effect of FBP1 as a tumor suppressor may also be mediated through enhancing the production of intracellular ROS (Figure 6). Our findings were consistent with the previously published data [8] showing fructose-1,6-bisphosphatase mediates cellular responses to DNA damage and aging in Saccharomyces cerevisiae. But how ROS exert cytotoxic and proapoptotic functions that would limit tumorigenicity and malignant progression, it was proposed that changes in cellular redox homeostasis and ROS levels will affect viability through redox modulation of the mitochondrial permeability transition pore opening leading to cytochrome C release, apoptosome assembly, and activation of executioner caspases, if cellular ROS levels reach a certain threshold incompatible with cellular survival [7], [14].

In summary, we found that FBP1 is frequently reduced by promoter hypermethylation in most liver and colon cancer cell lines and primary tumor tissues. Our results suggested that epigenetic inactivation of FBP1 was an important factor in human liver and colon carcinogenesis. We also demonstrated that promoter hypermethylation-mediated silencing of FBP1 could be reversed by pharmacologic demethylation and restoration of FBP1 suppressed tumor cell growth through inducing G2-M phase cell cycle arrest and an increase in ROS generation. Therefore, it will be valuable to explore the possible application of FBP1 as a molecular marker for the detection and treatment of these malignancies.
